# From roadmap to a sustainable end-to-end individualized therapy pathway

**DOI:** 10.1177/26330040251339204

**Published:** 2025-05-27

**Authors:** Anneliene H. Jonker, Elena-Alexandra Tataru, David P. Dimmock, Alison Bateman-House, Holm Graessner, Gareth Baynam, Erika F. Augustine, Adam Jaffe, Anna M. G. Pasmooij, Oxana Iliach, Richard Horgan, James Davies, Shruti Mitkus, Larissa Lapteva, Matthis Synofzik, Timothy W. Yu, Daniel O’Connor, Annemieke Aartsma-Rus

**Affiliations:** Health Technologies and Services Department, Techmed Centre, University of Twente, Enschede, The Netherlands; Fondation Maladies Rares, Paris, France; Creyon Bio Inc, San Diego, CA, USA; Division of Medical Ethics, Department of Population Health, Grossman School of Medicine, NYU Langone Health, New York, NY, USA; Centre for Rare Diseases, University Hospital Tübingen, Tübingen, Germany; Rare Care Centre, Perth Children’s Hospital, Perth, Australia; Kennedy Krieger Institute, Baltimore, MD, USA; School of Clinical Medicine, Faculty of Medicine and Health, University of New South Wales, Sydney, NSW, Australia; Dutch Medicines Evaluation Board, Utrecht, The Netherlands; Division of Pharmacoepidemiology and Clinical Pharmacology, Utrecht Institute for Pharmaceutical Sciences (UIPS), Utrecht University, Utrecht, The Netherlands; Certara, Toronto, ON, Canada; Cure Rare Diseases, Woodbridge, CT, USA; MRC Molecular Haematology Unit, University of Oxford, Oxford, UK; Global Genes, Washington, DC, USA; US Food and Drug Administration, Silver Spring, MD, USA; Division of Translational Genomics of Neurodegenerative Diseases, Hertie Institute for Clinical Brain Research, University of Tübingen, Tübingen, Germany; German Center for Neurodegenerative Diseases (DZNE), Tübingen, Germany; Division of Genetics and Genomics, Boston Children’s Hospital, Harvard Medical School, Boston, MA, USA; The Association of British Pharmaceutical Industry, London, UK; Department of Human Genetics, Leiden University Medical Center, Albinusdreef 2, 2333 ZH Leiden, The Netherlands

**Keywords:** data sharing, education, individualized therapies, N-of-1 therapy, patient engagement, payment models

## Abstract

The field of individualized, or N-of-1, therapy development is growing and increasingly gaining attention as a novel option for people with serious diseases, caused by unique genetic variants for whom approved therapies are not available. The N-of-1 taskforce of the International Rare Disease Research Consortium previously outlined a roadmap of aspects involved in N-of-1 therapy development and implementation. Here, this follow-up paper looks forward and reflects on how to address existing gaps to advance the current state of individualized interventions toward an integrated and sustainable treatment development model. It discusses what needs to be established for N-of-1 therapies to be developed and utilized at a larger scale, which involves features like sustainability; safety; efficacy; regulatory aspects; dedicated registries and data sharing; tools; long-term treatment monitoring; partnering with patient advocates; and reimbursement models. It closes with recommendations to shape the future of individualized therapies, focusing on ethical implications, education, creation of tools, incentives for data sharing, and innovative payment models.

## Plain language summary

Creating a sustainable, individualized therapy plan

This paper discusses how to improve and expand the development of individualized treatments, known as N-of-1 therapies, for people with rare diseases caused by unique genetic variations. These are patients who often have no approved treatments available. The authors build on earlier guidelines for creating these therapies and explore ways to make them more sustainable and scalable. Key topics include ensuring the safety and effectiveness of treatments, creating systems for data sharing and patient registries, partnering with patient advocates, and developing new payment models. The paper concludes with suggestions for addressing ethical concerns, supporting education, encouraging data sharing, and creating incentives for the future of individualized therapies.

**Limitations:** While the roadmap is suitable for different kinds of N-of-1 therapies, some variations might not be clarified further in the paper.

## Introduction

Developing therapies for people living with untreatable rare diseases is an unmet public health need, impacting children and adults,^1–3^ with a share of these individuals having genetic aberrations that are very rare or (almost) unique. Worldwide, over 350 million people are affected with a rare disease, being defined as occurring in less than 1 in 2000 individuals, and as such statistics indicate that a number as high as 1 in 20 people could be affected by a rare disease.^4,5^ N-of-1, or individualized, therapies are increasingly gaining attention as a possible solution for individuals with extremely rare pathogenic variants, for whom traditional commercial treatment development paths are considered unlikely.^6^ For this perspective paper, we define an N-of-1 approach as an individualized therapy developed for and used by one or very few people living with a rare disease. N-of-1 therapies are different from N-of-1 trials, where one or more drugs targeting a disease symptom and a placebo are compared in a single individual in a crossover design.^6^ The N-of-1 therapy development or individualized treatment development is aimed toward continuous treatment (although currently still in an experimental setting), focused on the well-being of the single patient.

In this new approach, interventions for individuals with a genetic disease target, specific, causative genetic variants, addressing core disease pathobiology. Over the last 5 years, a growing number of individualized treatments of different therapeutic modalities, including but not limited to antisense oligonucleotides (ASO), gene therapy, small molecules, and personalized cancer vaccines, have been developed and used in N-of-1 cases for individuals with severely debilitating and/or life-threatening genetic disease.^7–13^ Individualized treatments based on other therapeutic modalities, such as genome editing, are in development. The development of an N-of-1 treatment differs radically from traditional drug development, which can take up to a decade from proof of concept to first in human use. N-of-1 treatments necessitate a faster pathway, as compared to traditional orphan drug development for treatment of a condition, where clinical trials are set up to investigate the drug for larger prospectively defined cohorts of patients. For N-of-1, they need to be delivered before the patient succumbs to the disease or its progression reaches a stage where no benefit can be expected—similar to clinical trials, where enrolling patients unlikely to benefit is also avoided. Attempts to expedite non-clinical proof-of-concept and safety studies and manufacturing are underway; however, a clear end-to-end pathway of what is involved in N-of-1 treatment development has been lacking and the various efforts underway currently are not always globally aligned. Most importantly, from research ethics and public health perspectives, while each N-of-1 candidate treatment and its development is unique, learnings can be distilled from each case—regardless of whether they are considered objective successes or failures. If documented, shared, and analyzed this will facilitate not only the development of additional N-of-1 interventions for patients with the same disease but also such therapies in general. In this respect, N-of-1 treatments might be considered individualized interventions, as much as medicines themselves.^14^ The opportunity for iterative learning, however, critically depends on processes to facilitate data sharing. Capturing data on preclinical efficacy and safety, and clinical efficacy and safety evaluations is crucial in sustaining and scaling N-of-1 treatment development in addition to justifying payer reimbursements, widening access to eligible patients, and overcoming current concerns about inequitable access.

**Figure 1.**
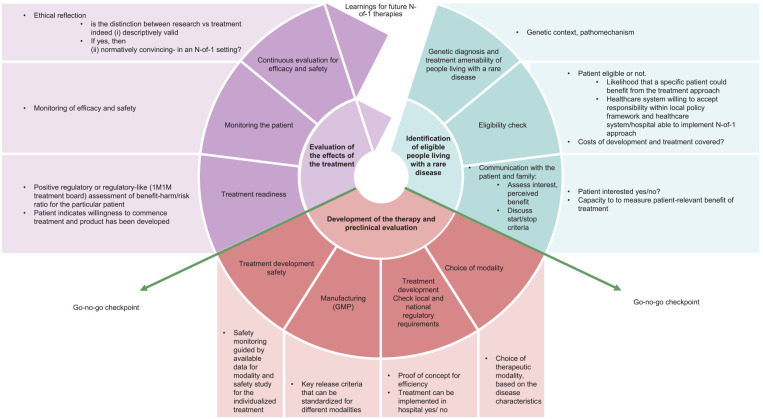
The roadmap of individualized therapy development.

The International Rare Diseases Research Consortium (IRDiRC) N-of-1 Task Force developed a “roadmap”—that is, a detailed plan to guide progress toward a goal—for the development of N-of-1 candidate therapies.^15,16^ This roadmap identifies actions and the information necessary to be obtained prior to the next step in the sequence of drug development and its delivery to individual patients (Figure 1). This perspective is grounded in the present but is forward-looking, to identify and address existing gaps needed to advance the field of individualized treatments toward an integrated and sustainable treatment development model.

An overview of the roadmap of individualized therapy. It includes three major stages: the identification of eligible people living with rare diseases; the development of the therapy and preclinical evaluation; and the evaluation of the effect of the treatment. It also includes go/no-go checkpoints, based on Jonker et al.^16^

## The setting for developing individualized therapies

Most of the individualized therapies developed and used to date are based on existing technologies and products already approved by regulators. For example, the two leading technologies that are currently used in making individualized therapies are ASOs and adeno-associated viral vectors (AAVs), which have both undergone non-clinical and clinical testing in patient populations, resulting in regulator-approved products such as SPINRAZA^®^ (nusinersen) and ZOLGENSMA^®^ (onasemnogene abeparvovec) for spinal muscular atrophy and QALSODY^®^ (tofersen) for SOD1-amyotrophic lateral sclerosis (ALS).^17–19^ To this end, further development of sustainable models and future infrastructure supporting individualized therapies should consider aspects of manufacturing, for example, freedom to operate to produce an N-of-1 version of an approved treatment and/or publicly available information on manufacturing.

## Treatment versus research?

There is an ongoing debate about whether N-of-1 therapeutic attempts are medical practice (i.e., the primary intent is to benefit the patient) or research (i.e., their primary intent is to collect generalizable data) or some intermediary entity (herein referred to as innovative practice). Consensus seems to be swinging toward the notion that N-of-1 therapeutic attempts based on well-established platforms as described above are innovative practices; in other words, they are experimental interventions primarily efficacy to benefit the patient, but which require some data collection in order to evaluate the safety and effectiveness of the intervention over time. What may seem a terminology dispute has serious implications because there are different regulations for the oversight and regulation of medical practice and research. However, there are no commonly agreed-upon international rules for overseeing innovative practice in this space. Navigating the apparent dichotomy between treatment versus research—for example, by the concept of “innovative practice”—is, however, needed not only for regulatory, for example, whether they are FDA approved or remain in perpetual IND, but also for payer and ethical reasons.^8^ Another challenge is data sharing and patient privacy. N-of-1 treatments are by definition not anonymous. It is up to the patient/surrogate decision-maker whether data are shared or kept private. To advance the field, data sharing is obviously preferred, and the reasons for doing this should be discussed with them from an early stage. The data from multiple independent N-of-1 treatments can, however, be presented at an aggregate level.

## Missing elements in the individualized treatment pathway

As the traditional commercial drug development pathway is not currently suitable for N-of-1 candidate therapies due to the lack of return on investment, there is a growing realization among stakeholders that there should be a novel approach to develop and manufacture these interventions, especially given the desire for scalability and sustainability.^14^ The current steps involved in individualized treatment development are shown in Figure 1. There are aspects of these steps that can be streamlined, and there is infrastructure that needs to be built and processes that need to be developed to ensure everyone who is eligible for an individualized treatment is able to benefit. Creating a novel intervention requires not only developing but also implementation processes to ensure proper administration, monitoring recipients for (potential) efficacy and safety, and identifying and informing potential recipients. This needs a change from the current system, wherein only a few people with a unique variant have been able to access candidate individualized therapies, due to the pathogenic variant or the type of technology. In addition, comprehensive and secure data reporting and analysis of the aggregated data collected from different patients with different diseases treated with different N-of-1 treatments does not yet exist. As such, we here identify major aspects that are currently lacking or that need adaptation.

## Identification of eligible people living with a rare disease

Developing these treatments requires a detailed mechanistic understanding of the molecular consequence of an individual's specific genetic variant, the pathobiology of the disease, and the severity of the condition.^20^ With improvements in diagnostic technology, more and more genetic variants that lead to a disease are being identified. However, the pathway between disease pathogenesis and the ability to develop treatment is not straightforward and typically unclear without extensive further investigation. Currently, N-of-1 cases are evaluated on a case-by-case basis, mostly by academic researchers and physicians who are aware of individualized treatment options; there is no current standard or scalable approach linking molecular diagnosis to assessment for or development of individualized therapy. A centralized system where actionable genetic variants for (individualized) treatments are automatically flagged in diagnostic databases would be more equitable and would help enable intervention during the relevant therapeutic timeframe. These systems are being developed by the *N* = 1 collaborative (N1C) and other initiatives dedicated to developing and sharing knowledge about individualized therapies. Building and validating tools to identify actionable pathogenic variants will take time.^21–24^

Sometimes the decision whether a patient is eligible for an N-of-1 treatment is straightforward. However, the reality is often not clear-cut when determining a patient’s eligibility or the most suitable therapeutic modality is most suitable. Processes need to be built to support timely discussions with relevant experts and stakeholders. Here, the model built by European network 1 Mutation 1 Medicine (1M1M) could be used as an example.^24^ This involves a multistep model, where first a small group of experts assesses whether the variant in principle is eligible for (in case of 1M1M) ASO-mediated exon skipping. If the variant is deemed potentially eligible, a gene group panel is assembled, including expert clinicians and researchers to evaluate whether the specific genetic aberration resulting in the disease qualifies for treatment and whether the patient is likely to benefit. If so, a larger treatment board is convened, which consists also of ethics experts, patient representatives, and national and/or European regulatory representatives. Here, it is discussed whether the specific ASO to be developed would be expected to benefit the patient and, if so, how to monitor this. Start and stop criteria as well as potential risks and uncertainties of treatment are discussed. The treatment board can reconvene at set intervals after treatment initiation to assess whether stop criteria need to be invoked or redefined (with input from the patient). These processes or some of their elements can be easily adapted to discuss other therapeutic options or to discuss which option is most likely to lead to benefit.

## Developing the treatment

Once the best treatment modality has been agreed upon for an individualized patient, the treatment needs to be developed. The roadmap outlined in Figure 1 lists the specific steps and quality criteria in place currently. As the specifics of such standards will vary with modality, this is something that each subfield will have to develop. Notably, within the ASO field, proposals have already been made, outlining a tool to perform a quality risk assessment for a manufacturing process for a new ASO based on how similar it is to previously developed ASOs with regards to chemical modifications of bases, length, and purification techniques.^25^ Pioneering efforts in this space have originated in academia but have involved close partnerships with industry experts contract research organizations and drug manufacturers. A key example is the development of the first *N* = 1, an ASO for a patient with CLN7 Batten disease, which was coordinated by Boston’s Children’s Hospital in collaboration with representatives from ASO developers (e.g., Ionis Pharmaceuticals) ASO manufacturers (e.g., Chemgenes) and CROs (Charles River Laboratories).^26^ First, pharmaceutical companies have a wealth of expertise with drug development that a growing number are sharing as in-kind contributions to the *N* = 1 developers (see e.g., the website of the *N* = 1 Collaborative: https://www.n1collaborative.org/resource-hub). Secondly, specific aspects of development are performed often by specialized companies, for example, manufacturing and safety or toxicity study. Thirdly, a pharmaceutical company may hold intellectual property rights on the technique that is proposed to be used in an individualized setting. Given that individualized treatments are currently not commercially profitable, it is hoped and anticipated that companies will provide licenses to groups that are developing an individualized treatment or establish and maintain manufacturing platforms with capabilities to make individualized therapies upon need. If individualized treatment development moves from a case-by-case development to a more streamlined platform-type of development for a given modality, it is likely that this will be taken over by a company, as this approach fits less with the typical academic research setting and allows for production at scale for multiple patients. However, this will hinge on whether there is a sustainable model for reimbursement of the developed individualized treatments,^15,27^ as described later in this paper.

## Communication with patients

Processes and tools are needed to allow optimal consent discussions with the patient and/ or their family when an individual is potentially eligible for an individualized treatment. Informed consent documents that clearly and comprehensively outline the particulars of individualized treatments with regard to treatment burden, risks, uncertainties, and potential benefit would facilitate discussion between clinicians and candidate patients and/or their families. Even before any formal consenting process begins, attention needs to be paid: informing a patient/family of the possibility of an individualized treatment too early, at least without very clear caveats, can lead to disappointment if it turns out the patient is ultimately ineligible. However, informing a patient/family with some considerable time in advance to be able to evaluate and weigh the burdens, risks, uncertainties, and potential benefits is vital and argues for informing them of this option earlier, rather than later, in the process. This would also enable an ongoing dialogue in which the clinician may be able to assess whether the patient/family’s expectations for possible benefit are realistic and to start collecting patient data so there will be before and after treatment initiation trajectory data. It is important to inform patients and/or families that learnings from failures or unexpected adverse events are crucial for future advancements and for the benefit of future patients. For this reason, long-term follow-up and, when applicable, post-mortem analysis are essential for assessing the overall impact and outcomes of e the procedure. For some individuals, targeted therapy may not be successful or may not prevent continued disease progression. In the event of death, for certain fatal diseases, particularly those involving cardiac or central nervous systems, autopsy may be necessary to determine treatment effects—but also to help with generalizable learnings (e.g., on tissue distribution and differential neuronal uptake of the ASOs). Discussions about long-term follow-up should take place before the treatment is started, as these may impact willingness to participate. This should also entail discussions on the possibility that a treatment is not successful and might lead to the patient passing, what the patient’s wishes are regarding a potential autopsy. Having these discussions ahead of time is part of informed consent, and discussing the possibility of autopsy as a future possibility is vastly preferred over having to raise this topic for the first time upon a patient’s demise.

## Education

There is an increasing need for health literacy for patients, and the current overflow of information has further stimulated the need for reliable information. We live in the era of “Doctor Google” and we are entering the era of Artificial Intelligence (e.g., heralded in a lay-friendly version as ChatGPT) as a tool to collect, process, and disseminate information that none of us was able to process and access before. As such, it is becoming critical to educate different stakeholders, and, particularly, patients and health care providers to be able to understand benefits and risks of the therapy, evaluate potential of success for individual N-of-1 treatment and make an informed decision. Education is critical in achieving these goals, but even more critical is to use a reliable educational resource, such as Health Authorities websites and/or patient organization websites.^28–32^ While looking for educational material, it is important to ensure that information is reliable, scientifically proven, and non-biased. Here, disease-specific and umbrella rare disease patient advocacy groups are leading the charge in selecting and screening educational resources for people with rare diseases, and provide a trustworthy source for information aimed at creating informed and engaged patient communities as well as well-informed healthcare professionals.

As new models and the infrastructure for manufacturing and clinical applications of individualized therapies emerge, professional training, coordination, and open channels for communication among stakeholders will be critical for continued progress in this field. Dedicated registries collecting and sharing data on individual patients’ experiences may become excellent platforms for communicating successes and failures, observations and discoveries, and for enabling learning and education of treating physicians and patient communities at large. International organizations such as IRDiRC, NORD, ERDERA, and EURORDIS, among others, may promote information exchange and availability of tools useful in individualized therapy development. Such tools may include catalogs with matching genotypes, diseases, and available technologies with industrial platforms and laboratories capable of producing actionable treatment options. In addition, other public-facing communications from academic institutions and centers may contain references to available tools and protocols for measurement of treatment outcomes as well as methods and venues for reliable and scientifically sound non-clinical testing. Different sources of communication and information distribution may be used, including, for example, registry newsletters, regulatory and rare disease organizations’ websites with educational modules, and medical and chemical/biotechnology professional organizations’ educational courses to improve knowledge and information sharing about individualized therapies.

## Partnering with patient advocates and families

Patients, caregivers, and advocates have been, and remain, at the center of traditional orphan drug development.^40–44^ They are essential in different steps throughout the development process,^33^ such as helping to set research priorities, and—in the research design and planning phase—reviewing protocols for clinical studies, which allows for patient insights into clinically meaningful endpoints, and what risks may or may not be considered tolerable.^34,35^ Importantly, patient advocates also play an important role in sharing information with their disease community, providing disease-specific information to caregivers and co-partnering with their healthcare team on the most appropriate treatment options.^36–42^

Almost more so than for traditional orphan drug development, in the field of N-of-1, the role of people living with a rare disease and their carers is central to development. First, they need to be willing to be involved in the N-of-1 therapy development process, in which the field is rapidly evolving, and lessons are learned from each case.^14^ As a result of increasing health literacy and available information, patients and/or their families are sometimes driving these approaches themselves, meaning that they are identifying and approaching a researcher who can potentially develop an individualized therapy and raising money to support the development and access aspects. In the past, we have seen specific cases of N-of-1 development, which were a collective process where the different stakeholders, including the people living with the rare disease and their carers, worked hand in hand to make the N-of-1 therapy a reality.^7,45^ As the field is developing, patient advocates, rather than individual people living with a rare disease and their carers, will need to take up a position, in building a community for people with unique pathogenic variants. This could be part of existing nationwide or global patient organizations, dedicating a part of their efforts to individualized patients, building upon the experience of the first families having undergone individualized therapies. As such, they can build up expertise in advocating for the development, and assisting in the communication about, individualized therapies, also helped by processes developed by academic networks on patient eligibility and treatment readiness.^45^ In addition, at present, a part of the funding of the development of individualized therapies is currently provided by patients and carers. It is worth noting, however, that just as patients with unique variants may have difficulty finding appropriate specialists for medical care, they may have difficulty finding an appropriate patient advocacy “home,” given the specificity of many such groups and the difficulty of establishing such a group for a variant that impacts almost nobody other than themselves.

## Post-treatment care and inpatient monitoring

Most individualized treatments require long-term treatment and/or long-term monitoring, placing demands on the health care system. These include clinician time for treatment administration, access to a surgical room if needed, continuous in-depth assessment of a wide range of safety and efficacy outcome measures, coordination of all visits, and analyses of monitoring outcomes. Given that there is a hope for an anticipated increase in scale regarding the number of patients receiving individualized therapeutics, plans to manage cost are a critical issue for families and hospital systems, although an increasing number of individualized therapies will also likely drive efficiencies of critical mass.^43^ To make individualized treatments sustainable, funds are required not only the development costs but also the maintenance costs of the treatment. Whether this funding would come from hospitals, health insurance, governments, philanthropy, or a mix thereof is a complicated and still open question.

## Regulatory oversight and input

Regulatory authorities across the globe are tasked with protecting public health through overseeing clinical research with investigational products and providing marketing authorizations for new medicines with a positive benefit–risk balance. These approval processes were designed with cohorts of patients in mind; as such, the individualized setting might not be fully adapted to the current regulatory processes and frameworks. Innovative approaches are needed that recognize the unique aspects of N-of-1 therapy development, time-sensitivity, and the potential innovative yet developing science and technology. Regulators are increasingly making commitments to re-focus their efforts on proportionate regulatory function, prioritizing safety whilst recognizing innovation and timely access in areas of unmet medical need. The individualized therapy community welcomes the ambitions of initiatives such as the UK Rare Therapies Launch Pad (RTLP) and the US Food and Drug Administration (FDA) Rare Diseases Innovation Hub.^31,44–46^ These and other initiatives should work together and exchange information to ensure that regulation is not a barrier to access but rather an enabler.

One approach to enhance drug development is the utilization of platform technology, which allows developers to use the same methodology, process, and/or equipment to manufacture different products—provided they are similar enough as set out by the regulators. In May 2024, FDA issued a guidance document on its Platform Technology Designation program, with clearly defined eligibility and incentives.^47^ Additionally, platform technology has been included in the European Commission’s proposal for the revised draft EU pharmaceutical legislation.^48^ However, the EC proposal focuses on a marketing authorization approach for a medicine comprised of a fixed component and a variable component (“platform technology”), whereas the FDA’s platform technology designation program offers a designation specific to a manufacturing method rather than a direct marketing authorization. This FDA platform technology designation can then subsequently be used in the marketing authorization for a new medicine, likely resulting in a more efficient review process for that application. This difference in the regulatory definition of platform technology between the EC proposal and FDA guidance is confusing for stakeholders and would benefit from international alignment. Interest in platform technology is further exemplified by the European Medicines Agency’s Quality Innovation Group meeting of November 2024, which specifically addressed platform technology, including platforms for the manufacturing of personalized or individualized medicines, for example, covering different ultra-rare orphan indications.^49^ Although the individualized setting may not fully align with current regulatory processes, a platform technology approach used to promote innovative manufacturing and a marketing authorization approach may permit data pooling from patients treated with individualized therapies manufactured using the same platform technology.

A comparison can also be made to custom-made devices—medical devices used for the treatment of a health issue, that use a similar common element, but are customized to each patient. These custom-made devices collectively request regulatory approval for the therapeutic shared common element, yet can be used in medical practice in a customized manner without additional procedures.^50,51^ Likewise, for individualized treatment development, accepted quality standards or thresholds, defined by regulators, could be in place for manufacturing aspects for a given therapeutic modality to streamline and accelerate therapeutic development.

## Sharing and reiterative learning through dedicated registries and data sharing

As mentioned throughout this perspective, sharing of data, experience, and processes is crucial to scale up the development of individualized treatments. This starts with raising awareness of who is developing what. Groups embarking on the development of a specific individualized treatment ideally should be entering them by starting with an accessible catalog. This will prevent duplication of efforts and will also facilitate knowledge exchange of individualized treatments when additional eligible individuals are identified. Furthermore, it will facilitate collaboration between groups working on the same gene or disease to share protocols and, if needed, samples. This “individualized treatment catalog” will likely also result in screening for specific genetic sequences once individualized treatments are available. An example is atipeksen, an ASO developed for an individual with ataxia telangiectasia, carrying a variant that causes cryptic splicing—a variant type that is often overlooked.^10^ Currently, a second individual was identified to carry the same variant and now is being treated with the same ASO. Given that some types of genetic aberrations are underdiagnosed, it may bring to light that what was thought to be a unique variant is, in fact, a (relatively) common variant. In such cases, transitioning from treatment in an individualized setting to the traditional clinical development of a marketed therapy may be considered, as was the case with jacifusen.^52^ Collaboration between individualized treatment developers and those working on more common disease/genetic variant studies will help advance access to novel treatments.

While each individualized treatment is, by definition, individual, there will be learnings that can be applied at a larger scale from each case, particularly for so-called platform technologies.^47,53–55^ With each N-of-1 candidate treatment, there is an ethical imperative to document, share, and analyze data from that case. In this way, while each individual case may (or may not) have helped an individual patient, the gradual accumulation of learnings will advance a societal good, for example, the development of additional N-of-1 interventions. As such, it is essential to have an infrastructure in place to capture and share information. While this infrastructure as such is not globally available yet for individualized treatment development, the field does not have to start from scratch but instead can benefit from similar efforts that have built data-sharing infrastructures.^56–58^ One potential and reasonably developed approach to creating this infrastructure would be the establishment and maintenance of a large multinational registry for individualized therapies. Any party who can make a product or who owns an approved technology platform should be invited to participate in the registry to share data and contribute their experiences with individualized therapies. It is not new for biotechnology and pharmaceutical companies to have protocols that are separated from product development programs or to establish registries in which data are accumulated on the use of their products in “one off” cases (e.g., for post-approval drug surveillance programs). Similarly, academic researchers are used to collecting data on patients they treated in experimental settings. Separately, each participating party may have their own registry collecting data on manufacturing, testing, and use of individualized treatments that they made. Collectively, data from these participants can be valuable for their respective regional and national registries and, potentially, to contribute to a larger multinational registry. The multinational global registry will enable sharing a catalog of available technologies and maintaining scientific high-quality data pools but will also permit using separate region- and setting-specific mechanisms for reimbursement related to individual product making.

An example where many aspects may be adopted by the individualized treatment community is the European Society for Blood and Marrow Transplantation (EBMT), established in 1974.^56^ The EBMT arose in response to the growing need for a collaborative platform among medical professionals and researchers involved in the then-emerging field of bone marrow transplantation. The organization was founded by a small group of pioneering physicians and researchers who recognized the importance of sharing knowledge and data related to bone marrow transplants. The primary goal was to improve patient outcomes by pooling experiences and standardizing practices across Europe, after the first long-term successful bone marrow transplant in 1968, by Dr E.D. Thomas. One of their most important instruments is the EBMT registry, which was set up to systematically keep records of bone marrow transplant patients and pool data from multiple centers so that knowledge could be shared.^57,59^ By allowing these data to be analyzed, best practices were identified, complications were tracked, and domain-specific research was advanced. There are things that can be extrapolated from this effort to the individualized treatment community: from transplant and follow-up to the need to collect follow-up data after treatment is initiated in individuals; from matching a donor and a host (matching an eligible patient to a treatment developer), to matching additional eligible patients to a treatment that is available for a single individual.

## Data collection and monitoring across indications

Capturing data from individual N-of-1 candidate treatment efforts is crucial in sustaining and scaling these efforts through payer reimbursements, thus widening access to eligible patients and overcoming current justice concerns about inequitable access to these expensive endeavors. Yet data collection is also accompanied by ethical, legal, and practical concerns. The same may well be true of N-of-1 data without the establishment of early norms, standards, and expectations for the nascent field. Schoenmakers et al.^58^ have described a framework for multistakeholder patient registries in the field of rare diseases with a focus on neurogenetic diseases. Some conclusions and orientation can be drawn for data collection and monitoring across indication for the N-of-1 field.

- Considering the limited number of individualized therapy developments and treatments as well as the substantial deviation from the traditional cohort-driven drug development and post-marketing surveillance paradigm, data collections (in particular) of high-quality patient data for N-of-1 therapy developments and treatments should be done in the same federated or centralized registry setup worldwide, be multipurpose and thus serve different stakeholders including patients and families, clinicians, researchers, regulators, payers, and pharmaceutical industry. Although, these different stakeholders have different interests and expectations, they all profit from high-quality data in the same registry setup with regard to the various purposes they follow including research and knowledge gain, patient selection, natural history study, treatment planning and conduct, regulatory, reimbursement decision-making as well as monitoring outcomes and cost-effectiveness in the real world. Data quality needs to be a key focus from the very start and based on robust data lifecycle-encompassing procedures.
- An international consensus procedure involving multiple stakeholders to determine which data are to be collected in the registry, how these should be collected, and how data quality should be assured, as planned and to some extent already started in N1C and 1M1M^21,24^ may be resulting in indication overarching core and specific data items. Ensuring adherence of core elements with international standards and ontologies will enable linking the collected data with existing disease-specific disease registries while ensuring privacy. Integrated patient-reported outcome measures (PROM) in these registries could be used across indications to serve health economic purposes and provide indication-specific outcomes to ensure clinical relevance. Given that the N-of-1 field is highly dynamic, the requirement of balancing clear research objectives and retaining flexibility should be guiding the definition of data elements, the governance model, and the IT infrastructure.

The oversight of the N-of-1 data collection should be ascertained by a transparent governance model, which allows for data (re-)utilization as well as timely and safe data access by various stakeholders. To ensure this, a broad independent (without commercial interests) ownership for example by a network of academic institutions such as N1C or 1M1M might not only ensure access by the respective stakeholders but also ensure sustainability. An early involvement of the relevant stakeholders such as patient organizations, industry, regulatory, and Health Technology Assessment (HTA) agencies can be formalized in a consortium agreement that also addresses global data sharing and processing as well as biomaterial transfer. The Metachromatic Leukodystrophies Initiative (MLDi) has developed and implemented a respective consortium agreement that could serve as a blueprint for the N-of-1 field.^60^ Further key aspects of MLDi that might support N-of-1 data management are (i) placing data collection, analysis, and (re-)use in a comprehensive ecosystem that links diagnosis and newborn screening, healthcare services delivered by multidisciplinary boards, and actual treatment of MLD patients with research, as well as (ii) designing and implementing MLDi from the very beginning as a European and international organization in terms of network and legal framework compliant with different national regulations. Key from the governance perspective is that collected data are quickly accessible, independent, and trustworthy. To motivate and ensure data are being shared in this registry setup, there is a need to strengthen the obligation for registry data sharing thus ensuring improved accessibility of treatment development data to all stakeholders (with the right patient privacy safeguards in place), for possible re-use and optimization of future advancements.^61^

As with other data capturing and sharing efforts in the rare disease community, the N-of-1 therapeutics community must bear in mind the importance of FAIR (Findable, Accessible, Interoperable, Reusable) data principles in maximizing the utility of this precious patient-derived data.^62^

## Toward a sustainable funding and reimbursement model

Treating one person with a rare disease via the development of a N-of-1 therapy, clinical implementation, and follow-up currently comes at a significant cost. At present, there is no consistent way by which to pay for developing and accessing individualized treatments. Some efforts are funded by crowdfunding, others through philanthropy or by funding from the government, institutes, or departments. Moreover, a combination of funding sources, including government grants, institutes, non-profit organizations, and industry partnerships, constitutes a robust support network for the development of individualized therapy development. The Ultra-rare Gene-based Therapy (URGenT) network can be seen as one of the examples of individualized therapy development, illustrating collaborative efforts between academia, government agencies, and industry stakeholders to accelerate the translation of research into clinical practice.^63^ Institutes and non-profit organizations dedicated to rare diseases also contribute essential funding and resources, underscoring the collective commitment to addressing unmet medical needs.

However, the development and administration of individualized therapies are currently more based on a one-off model, rather than a sustainable one. As such, this raises the question of what elements should be paid for by the healthcare system and government and what, if any, by the individual patient or private insurance depending on the region. The cost of an individualized therapy development can be disaggregated into different elements (Figure 1). Specifically, Table 1 portrays the cost of the constituent elements in the pathway and relevant payers.^64^

**Table 1.** Cost items and the stakeholder (organization) that pays for the cost (suitability can be country-specific).

Cost item	Paid by
Diagnosis and identification of the person with a rare disease with a unique variant	Healthcare system/insurance or out-of-pocket (patient/carer)
Research to develop the individualized therapy	Research grants/philanthropy/crowdfunding
Production of the individualized therapy	Research grants/philanthropy/crowdfunding/in kind contribution companies
Clinical implementation of the individualized therapy	Hospital delivering and monitoring the treatment, or out-of-pocket costs (patient/carer)

With progress in science and regulatory reform, there is a possibility that the cost of individualized therapies will significantly reduce with time and ongoing efforts, as is typically the case with newly introduced innovative procedures. This could be stimulated due to the building upon platforms of available and approved technologies, reducing the individual cost per patient without increasing the risk of making an ineffective or unsafe therapy. As such, there is a difference in the cost for the first individualized therapy for a specific therapeutic model and a specific disease and incremental individualized therapies, in which the therapy development can be based upon the learnings of previous product making and clinical use. However, in the meantime, the costs of therapy for each individual might still be significant per development. The first individualized ASO, Milasen, was reported to have cost approximately 2 million US$, much of which was covered by the hospital and philanthropy.^9,15,65^ Cases that have been performed and published later on have not always been transparent on the costs, but the estimated cost from the IRDiRC Task Force discussions has been reported between 1.5 and 2 million US$. At such an expense, and with such unstable funding, further individualized therapeutic development would have been limited to the lucky few able to pay or raise money for treatment efforts and also raises a significant ethical debate.^66^ Furthermore, alongside the costs for therapy, health systems should recognize the costs of not treating patients, who often require increasing health resources and care packages as their conditions progress.

This is a direct challenge to the roadmap’s goal of equity of access, in which all patients with a possibly treatable rare disorder can benefit from these therapies, regardless of geographical, cultural, and/or socio-economic backgrounds.^65,67,68^ An essential first step toward this goal is ensuring that patients are genetically diagnosed and educated about how to seek out an individualized therapeutic during their therapeutic window and that individualized therapies are developed and provided in an equitable manner across the world. Consequently, beyond reducing the cost per patient of such therapies, new funding or payment models are needed. Several countries are exploring new models for innovative therapies, for which traditional payment models are not successful.^69–71^ One potential model is the subscription payment model, where a subscription fee—ideally funded by a payor like the government or an insurance company—would support a Center of Excellence or a (non-)commercial organization.^15^ This revenue would enable the center to sustain ongoing innovation, streamline development processes, and fund critical projects for patients with rapidly advancing disorders. Another model is the pay-for-performance model, where payments to providers are directly linked to specific performance metrics, such as patient outcomes.^71^ As N-of-1 therapy development becomes more established, there may be growing commercial interest in developing such therapies, beyond the current focus on companies providing services like technology use for product manufacturing, following good manufacturing practices (GMP), and initial non-clinical safety studies. Furthermore, pay-by-performance is currently being studied for cell therapy in Germany.^72,73^ Another reflection is the need to develop a payment appraisal and approval form, in which regulators and payers can evaluate the treatment under development, in several steps, while being able to have proper controls in check to ensure patient safety but also keeping the speed of development, due to the severe nature of these diseases.

## Recommendations and target audiences

As this field is developing, the Task Force (TF) has set out recommendations around six themes:

### General recommendations

The field of individualized therapies should be further strengthened by a clear positioning in the field of orphan treatments. The N-of-1 treatment development cannot be scaled up or sustained without ways to share data, experience, and processes.

### Education

With the further evolution of the field of individualized therapies, there is a need to develop specific educational materials, as a next step to some of the existing resources.^29^ These materials should be developed for categories of stakeholders, such as people living with rare diseases, physicians, therapy developers, including multidisciplinary biotechnology professionals, regulators, laboratory scientists, geneticists, and ethicists.

### Creation of tools to deliver the pathway

In this and previous work, we have shaped an initial roadmap for the development of individualized therapies. However, these are primarily the main steps and potential stakeholders involved in those steps. A further mapping of the different tools that are needed for each of the steps needs to be done.

### Final map of what the pathway looks like

The current roadmap is based on the current knowledge and tools (Figure 1). However, likely with future development, the roadmap will be adapted and fine-tuned in the future. The roadmap should be adapted regularly based on new developments in the field.

### Partnering with patient advocates and families

Working with patient advocates and families and engaging them in an early stage is essential for the field of individualized therapies. It is recommended that there are specific patient group setups for patients with unique genetic variants.

### Implement (positive) incentives for data sharing

The only way to align and improve is to actively collect and share data. There may be ways to create positive financial incentives, incentives dealing with academic credit/promotion/eligibility for grants/ability to publish. Equally or perhaps even more important is the need to develop a system of norms by which the field operates.

### Developing innovative payment models

There is a high need to develop novel, innovative payment models for individualized therapies. This is critical to unlocking their full potential and ensuring that these therapies are available to those who need them most. By adapting payment structures, incorporating innovative models, addressing uncertainties, and fostering global collaboration, we can create a healthcare system that supports the continued development and accessibility of individualized medicine. As such, there is a need to organize meetings with all stakeholders, to develop new reimbursement models, building upon current innovative payment models, but also to continue the ethical discussions that are needed around the development of innovative payment models and equitable access.^28,69,74,75^ Critically, payment hinges on compelling data; as such, the evidentiary needs of payers must be taken into consideration when building systems for collecting, sharing, and analyzing data.

## Conclusion

Individualized therapy offers significant potential for transforming treatment, especially for rare diseases, but several challenges must be addressed for its success. Key steps include further positioning individualized therapies in the field of rare disease therapies, and developing a sustainable infrastructure and processes in a way that all who are eligible for an individualized treatment are able to benefit from it. Robust data sharing, standardized practices, and stakeholder education are also essential, as are dealing with the accompanying ethical and policy questions such as who has primary ownership over data, the patient, or others? Additionally, specialized tools for outcome measurement and cost analysis will optimize treatments and help develop funding models beyond current sources like crowdfunding and philanthropy. Finally, there is a need for fostering collaboration with industry. Ultimately, overcoming these hurdles will pave the way for personalized therapies to benefit patients globally.

## Appendix

### Abbreviations

1M1M 1 mutation 1 medicine

AAV Adeno-Associated Virus

ALS Amyotrophic Lateral Sclerosis

ASO AntiSense Oligonucleotides

EBMT European Society for Blood and Marrow Transplantation

FAIR Findable, Accessible, Interoperable, Reusable

FDA US Food and Drug Administration

GMP Good Manufacturing Practice

HTA Health Technology Assessment

IND Investigational New Drug

IPD Individual Participant Data

IRDiRC International Rare Diseases Research Consortium

MLDi Metachromatic Leukodystrophies Initiative

N1C *N* = 1 Collaborative

PROM Patient Reported Outcome Measures

RTLP Rare Therapies Launch Pad

TF Task Force

URGenT Ultra-rare Gene-based Therapy

## Declarations

**Ethics approval and consent to participate:** Not applicable.

**Consent for publication:** Not applicable.

**Competing interests:** A.A.R. is an unpaid board member of the Dutch Centre for RNA Therapeutics and an unpaid board member of the *N* = 1 collaborative. A.B.H. is an uncompensated volunteer for the n-Lorem Foundation’s Access to Treatment committee. A.J. has received speaking fees from Vertex Biopharmaceuticals. D.P.D. is an employee of Creyon Bio. H.G. is a Member of the Board of Directors of the *N* = 1 Collaborative. D.O.’C. is involved in the Rare Therapies Launch Pad as representative for the ABPI, a non-financial interaction. J.D. is co-founder of Nucleome Therapeutics and provides consultancy to the company, in addition, he has intellectual property licensed to BEAM Therapeutics and he receives revenue from this license and holds personal shares. M.S. has received consultancy honoraria from Ionis, UCB, Prevail, Orphazyme, Biogen, Servier, Reata, GenOrph, AviadoBio, Biohaven, Zevra, Lilly, and Solaxa, all unrelated to the present manuscript; and is an unpaid board member of the *N* = 1 collaborative. R.H. is CEO and founder, Cure Rare Disease; CEO and founder, Myofinity Biosciences; and Consultant, Neuromuscular Disease Foundation. T.Y. has received research support for an *N* = 1 trial in ataxia telangiectasia from EveryONE Medicines, and is named as an inventor on three patents pertaining to antisense oligonucleotide treatments: for transposon-associated diseases (WO2019055460A1), ataxia telangiectasia (US20230174979A1), and progranulin (US11359199B2). He has also received scientific consulting fees as pertains to ASO therapies from GeneTx, RegUp, and Servier Pharmaceuticals, serves as an unpaid Board Member for the *N* = 1 Collaborative, the Oligonucleotide Therapeutics Society, and the Society for RNA Therapeutics, and an unpaid scientific advisor to several rare disease foundations. A.H.J., E.A.T, G.B., S.M., O.I., E.F.A., A.M.G.P., and L.L. declare no competing interests.

**Availability of data and materials:** Not applicable.
